# Alterations in Lipid Metabolism and Hepatopancreatic Lipidomics Induced by Microcystin-LR Exposure in Common Carp (*Cyprinus carpio*)

**DOI:** 10.3390/ani15192803

**Published:** 2025-09-25

**Authors:** Haoyang Zhao, Mengya Lou, Xin Liu, Wenjun Wen, Xiaoyu Li

**Affiliations:** College of Life Sciences, Henan Normal University, Xinxiang 453007, China; zhaohaoyang@stu.htu.edu.cn (H.Z.); 18838713497@163.com (M.L.); lxxhh1281@163.com (X.L.); 18339501042@163.com (W.W.)

**Keywords:** microcystin, common carp, oxidative stress, hepatotoxicity, lipid metabolism

## Abstract

Microcystin-LR (MC-LR) is a hepatotoxin produced by toxic cyanobacteria such as *Microcystis aeruginosa* and it poses significant risks to aquatic organisms and human health. However, research on the long-term effects of environmental MC-LR exposure on lipid metabolism in fish is relatively scarce. This research investigates the effects and underlying mechanisms of chronic (one month) low-dose (3.5 μg/kg) MC-LR exposure in common carp (*Cyprinus carpio*) by using biochemical assays, histopathology, molecular analyses, and lipidomics. In this study, MC-LR exposure significantly altered serum enzyme activities and lipid profiles, induced hepatic inflammation and lipid accumulation, and disrupted hepatopancreatic structure. Meanwhile, key regulators of lipogenesis, fatty acid β-oxidation, and cholesterol metabolism were dysregulated, indicating enhanced lipid synthesis and impaired catabolism. Elevated oxidative stress and pro-inflammatory cytokines likely contributed to lipid metabolic disturbances, exacerbating the hepatotoxicity of MC-LR. Lipidomics profiling revealed significant disruptions in glycerophospholipids, glycerolipids, and sphingolipids, highlighting impaired lipid homeostasis. This study provides novel insights into the hepatic lipid metabolic disorders induced by MC-LR in fish.

## Simple Summary

Microcystin-LR is a kind of cyanotoxin. When cyanobacterial blooms occur, a large amount of cyanotoxin is produced, which poses ecological risks to aquatic organisms. In this study, we found that chronic exposure to low-dose Microcystin-LR caused oxidative stress, inflammatory responses, and hepatocyte damage. Meanwhile, the expression of genes and proteins related to lipid metabolism was disturbed. These findings deepen our understanding of the hepatotoxicity of Microcystin-LR and provide insights into hepatoprotective strategies in fish.

## 1. Introduction

The ongoing nutrient enrichment of aquatic environments across the globe has significantly intensified the occurrence and severity of cyanobacterial outbreaks in both natural lakes and aquaculture ponds [1,2]. Cyanobacterial blooms impair water quality and lead to the discharge of considerable amounts of cyanotoxins, which severely disrupt the ecological balance and functioning of aquatic ecosystems. Within the class of cyanotoxins, microcystins (MCs) are widely detected and extensively investigated [3,4]. There is growing scientific consensus that MCs can build up to hazardous concentrations in a variety of aquatic systems. For example, microcystin levels in Steilacoom Lake (USA) have been reported to reach 2.7 mg/L. In several African regions, concentrations commonly exceed 10 mg/L [5,6]. In China’s Lake Taihu, cyanobacterial blooms have resulted in microcystin concentrations averaging 11.8 μg/L, occasionally surging to 35.8 μg/L in peak periods [7]. Similarly, microcystin concentrations in aquaculture ponds have been observed to peak at 10 μg/L [8]. Microcystins are a group of potent hepatotoxins that exhibit significant toxicity to aquatic organisms and public health [9]. Moreover, these toxins also hinder the sustainable development of aquaculture and result in considerable economic losses [10]. Over 250 variants of microcystin have been discovered, with microcystin-LR (MC-LR) being of particular concern as it is highly toxic, chemically stable, and widely distributed in freshwater environments [11]. The toxicity of MC-LR is largely attributed to its suppression of protein phosphatases 1 and 2A (PP1 and PP2A), essential regulators of intracellular signaling and homeostasis [12]. The suppression of phosphatases leads to various cellular dysfunctions, notably oxidative stress, apoptosis, and inflammation, with hepatocytes being especially vulnerable [13]. Recent research has increasingly concentrated on the effects of MC-LR on lipid metabolic in hepatic tissues. Chronic exposure to low concentrations of MC-LR has been shown to impair hepatic lipid homeostasis, induce inflammatory responses, and influence transcriptional pathways linked to lipid anabolism and mitochondrial β-oxidation in mice [14]. Additionally, the toxicological effects of MC-LR include marked histopathological damage, lipid accumulation, and disruption of intestinal detoxification processes in tadpoles (*Lithobates catesbeianus*) [15]. Notably, sustained contact with environmentally typical levels of MC-LR has been correlated with lipid accumulation in the liver of mice [16].

Lipidomics is a high-throughput analytical approach that comprehensively examines alterations in lipid composition [17]. By profiling both lipid families and individual lipid species, lipidomics enables the efficient characterization of lipid alterations and their functional roles in various physiological and pathological processes, thereby it can provide valuable insights into underlying molecular mechanisms and biological activities [18]. Like other omics technologies, lipidomics captures dynamic shifts in lipid profiles in response to external stimuli, offering a comprehensive perspective on lipid metabolism and its regulation [19]. Moreover, lipidomic analysis can help elucidate the molecular basis of phenotypic variations and contribute to the understanding of disease pathology and responses to environmental toxicants [20]. Despite its significant potential in toxicological research, lipidomics has been underutilized in studies investigating the toxic effects of MCs.

Common carp (*Cyprinus carpio*) is widely distributed and of considerable economic importance, and it has been a key species in aquaculture in China [21]. Due to its sensitivity to environmental fluctuations, common carp serves as an effective bioindicator for assessing water quality and pollution levels [22]. Earlier investigations have largely examined how MCs contribute to liver damage, immune dysfunction, redox imbalance, and inflammatory responses in fish [23,24]. In the present study, we conducted lipidomics analysis alongside histological, biochemical, and molecular indices to examine the impact of MC-LR on hepatic metabolic homeostasis in common carp. This integrated strategy was designed to clarify how MC-LR affects lipid metabolic processes, thereby offering new insights into its toxicological effects on fish.

## 2. Materials and Methods

### 2.1. Reagents

MC-LR (purity ≥95%, Algal Science Inc., Taoyuan, China) was solubilized using acetone (Sinopharm, Shanghai, China). The solution was preserved at −20 °C. Isopropanol, formic acid, and ammonium formate (HPLC grade), while methanol and acetonitrile (analytical grade) were bought from Sigma (Shanghai, China).

### 2.2. Experimental Design

Common carp (20.1 ± 1.6 g) were sourced from a nearby fish hatchery (Xinxiang, China). Under controlled laboratory conditions, fish were housed in a 16:8 h light-dark schedule, with water temperature held at 25 ± 1 °C and pH regulated to 7.2 ± 0.2. Carp were fed twice daily at regular intervals using a pellet diet containing 32% protein (Tongwei Co., Ltd., Chengdu, China).

The experiment involved 480 fish. At the beginning of the study, 120 fish were randomly allocated to each sampling time point (3, 7, 15, and 30 d). For each time point, the fish were divided into two groups, each comprising three replicate tanks (20 fish per tank, 100 L). The treatment group (MC) was injected intraperitoneally (i.p.) with 3.5 μg/kg MC-LR every 3 d, while the control group (CK) received 0.9% NaCl at the same interval. The MC-LR injection dose was selected based on prior work in our laboratory, where it was determined to be 1% of the 24 h LD50 (357.08 μg/kg) for common carp [25]. At 3, 7, 15, and 30 d of exposure, six individuals were randomly chosen from each tank. Isolated hepatopancreatic tissues were preserved at −80 °C for subsequent analyses. For histological assessment, tissues from three individuals from each group were preserved using 4% paraformaldehyde. All animal procedures adhered to the ethical principles approved by the Ethics Committee of Henan Normal University, with approval date of 25 November 2024.

### 2.3. Histological Observation

Hepatopancreatic tissues were soaked in 4% paraformaldehyde and processed through an ethanol series for dehydration. The tissues were embedded in paraffin, cut into 5 μm sections, and stained with H&E staining. Frozen hepatopancreatic tissues were sectioned at 8 μm and stained with Oil Red O. Tissue morphology was investigated with an Olympus CX31 optical microscope (Olympus Corporation, Tokyo, Japan).

### 2.4. Gene Expression Measurement

Total RNA was extracted with TRIzol reagent (Takara, Dalian, China), and first-strand cDNA was synthesized using the HiFiScript kit (Cwbio, Taizhou, China). Quantitative PCR was performed using SYBR Green Mix (Monad, Suzhou, China). Primer sequences (Table S1) were synthesized by GENEWIZ (Beijing, China). Cycling conditions: Preheat at 95 °C for 10 min, then perform 40 cycles of 10 s at 95 °C and 30 s at 60 °C.

### 2.5. Biochemical Assay

Hepatopancreatic tissue were homogenized in ice-cold phosphate-buffered saline (PBS, pH 7.4), followed by centrifugation at 10,000× *g* for 15 min at 4 °C, and the resulting supernatants were collected for subsequent analyses. Aspartate aminotransferase (AST), alanine transaminase (ALT), alkaline phosphatase (AKP), total cholesterol (TCHO), triglycerides (TG), low-density lipoprotein cholesterol (LDL-C), and high-density lipoprotein cholesterol (HDL-C) in serum and malondialdehyde (MDA), catalase (CAT), superoxide dismutase (SOD) and glutathione (GSH) in hepatopancreatic tissue were measured using assay kits. Details are in Supplementary Methods S1.

ELISA kits were used to quantify reactive oxygen species (ROS), interleukin-1β (IL-1β), and tumor necrosis factor-α (TNF-α) in hepatopancreatic tissue, as described in Supplementary Methods S2. To evaluate comprehensive ecotoxicological effects, SOD, CAT, GSH, MDA, and ROS were integrated to calculate the Integrated Biomarker Response version 2 (IBRv2) index.

### 2.6. Western Blot

Hepatopancreatic tissue was analyzed by Western blot using a previously established method [26]. Peroxisome proliferator-activated receptor-α (PPAR-α) (#WL00978), cluster of differentiation 36 (CD36) (#WL02390), and GAPDH primary antibodies (#WL01114, dilutions: 1:500 and 1:1000, respectively) were supplied by Wan Class Biotechnology Co. (Shenyang, China). Sterol regulatory element-binding protein 1c (SREBP-1c) (#AF6283) were supplied by Affinity Biosciences (Changzhou, China). Beyotime Biotechnology (Shanghai, China) provided the HRP-conjugated goat anti-rabbit IgG (#A0352, 1:3000, *v*/*v*) used as the secondary antibody. ImageJ software (version Fiji 2.1.0) was employed to perform densitometric analysis and visualize the protein bands.

### 2.7. Lipids Extraction

Lipids were extracted from samples using a standard procedure. First, samples were vortexed (5 s), mixed with 240 µL pre-chilled methanol, revortexed (30 s), then supplemented with 800 µL methyl tert-butyl ether (MTBE) and sonicated (4 °C, 20 s). After 30 min room temperature incubation, centrifugation ensued (14,000× *g*, 15 min, 10 °C). The organic supernatant was collected and nitrogen-dried [27].

### 2.8. LC-MS/MS Methodology

The isolated lipids were dissolved in 200 µL of a 90% isopropanol–acetonitrile mixture and then subjected to centrifugation at 14,000× *g* for 15 min. Following this, 3 µL of the supernatant was taken for chromatographic examination. Lipid separation was carried out on a charged surface hybrid (CSH) C18 column (Waters, Milford, MA, USA) under reverse-phase gradient conditions. The chromatographic process utilized a 300 µL/min flow rate, with solvent A being a 6:4 mixture of acetonitrile and water, and solvent B consisting of acetonitrile and isopropanol in a 1:9 ratio. Formic acid (0.1%) and ammonium formate (0.1 mM) were added to both phases. The gradient started with 30% solvent B for the initial 2 min, then gradually ramped to 100% over 23 min, and the system was restored to 5% solvent B for 10 min [28]. The Q-Exactive Plus mass spectrometer (Thermo Scientific, Waltham, MA, USA) was used for the analyses [29].

### 2.9. Statistical Analysis

Data analysis and visualization were applied by GraphPad Prism 8.0 (GraphPad Software, San Diego, CA, USA). Normality and variance homogeneity were evaluated using Shapiro–Wilk’s and Levene’s tests. Two-way ANOVA detected significant differences (*p* < 0.05 and *p* < 0.01) between the mean values of the CK and MC treatment group.

## 3. Results

### 3.1. Increased Serum Enzyme Activity and Hepatopancreas Injury

**Figure 1:**
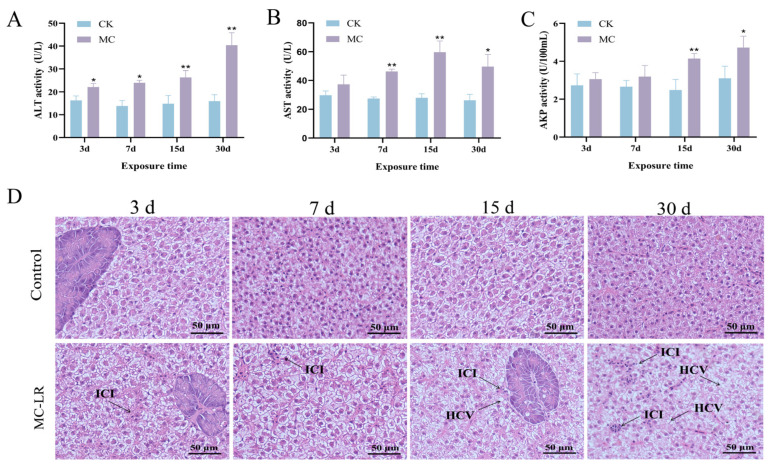
Effect of MC-LR exposure on serum enzyme activities and the structure of hepatopancreas of common carp. (**A**) ALT activity in serum. (**B**) AST activity in serum. (**C**) AKP activity in serum. (**D**) Representative histological images of hepatopancreases. Asterisks denote significant differences between the CK group and the MC group (* *p* < 0.05; ** *p* < 0.01). HCV: hepatocyte cytoplasmic vacuolization, ICI: inflammatory cell infiltration (magnification: ×400; Scale bar: 50 µm).

After exposure to MC-LR, a marked rise in serum ALT levels was detected from 3 d onward (*p* < 0.05) (Figure 1A). Serum AST activity also showed a significant elevation beginning at 7 d (Figure 1B). Furthermore, serum AKP activity increased significantly at 15 d (*p* < 0.01) (Figure 1C). Compared to the control group, hepatocytes exhibited cytoplasmic vacuolization and inflammatory cell infiltration at 30 d of MC-LR exposure (Figure 1D).

### 3.2. Altered Serum Lipid Levels and Hepatic Steatosis

**Figure 2:**
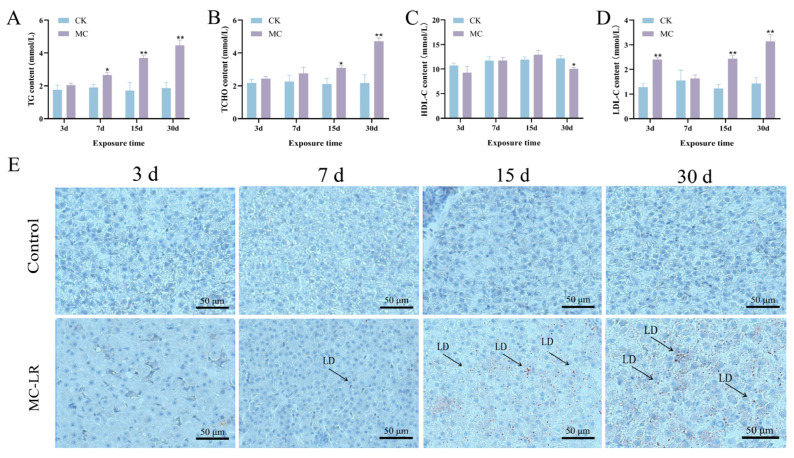
Effect of MC-LR on serum lipid levels and Oil Red O staining in the hepatopancreas of common carp. (**A**) TG content in serum. (**B**) TCHO content in serum. (**C**) HDL-C content in serum. (**D**) LDL-C content in serum. (**E**) Representative histological images of oil red O-stained sections. Asterisks denote significant differences between the CK group and the MC group (* *p* < 0.05; ** *p* < 0.01). LD: lipid deposition (magnification: ×400; Scale bar: 50 µm).

Serum TG levels showed a marked elevation beginning on 7 d of MC-LR exposure (*p* < 0.05) (Figure 2A), while serum TCHO levels showed a significant elevation at 15 d (*p* < 0.05) (Figure 2B). Furthermore, serum LDL-C levels elevated significantly in the MC-LR treatment group at 3 d (*p* < 0.01) (Figure 2C). In contrast, exposure to MC-LR caused a marked decrease in HDL-C levels at 30 d (*p* < 0.05) (Figure 2D). Oil Red O staining indicated excessive lipid deposition in fish hepatopancreas (Figure 2E).

### 3.3. Lipid Metabolism Disorder in Hepatopancreas

**Figure 3:**
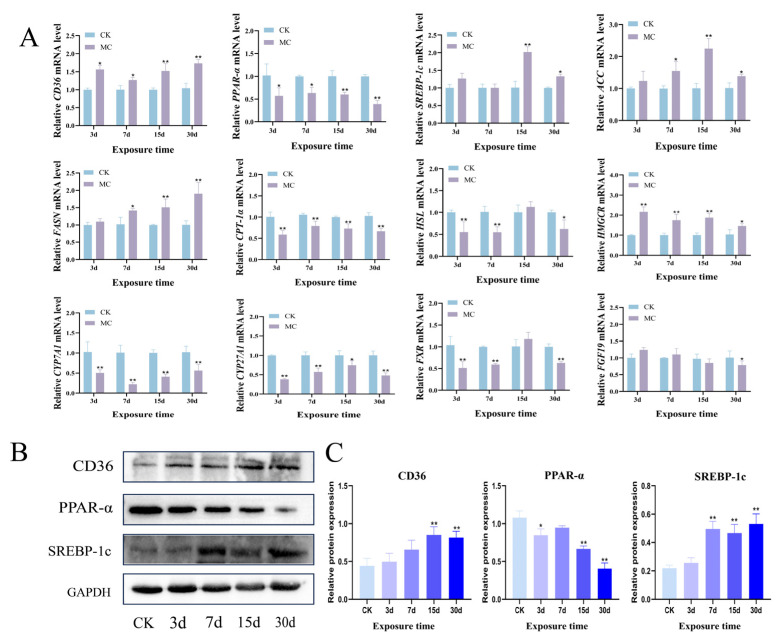
Effect of MC-LR on the expressions of lipid metabolism-related genes and proteins in the hepatopancreas. (**A**) Gene expression levels of *CD36*, *PPAR-α*, *SPERB-1c*, *ACC*, *FASN*, *CPT-1α*, *HSL*, *HMGCR*, *CYP7A1*, *CYP27A1*, *FXR*, and *FGF19*. (**B**) Protein levels of CD36, PPAR-α, and SPERB-1c. (**C**) Gray protein ration of CD36, PPAR-α, and SPERB-1c to GAPDH. Asterisks denote significant differences between the CK group and the MC group (* *p* < 0.05; ** *p* < 0.01).

As illustrated in Figure 3A, hepatopancreas *CD36* mRNA expression in MC-LR exposed fish showed a marked upregulation beginning at 3 d (*p* < 0.05). Meanwhile, MC-LR treatment significantly increased in *SREBP-1c*, Acetyl-CoA Carboxylase (*ACC*), and Fatty Acid Synthase (*FASN*) mRNA expression, with particularly high levels observed at 15 d and 30 d (*p* < 0.05). Furthermore, the expression of *PPAR-α*, carnitine palmitoyltransferase *1α* (*CPT-1α*), and hormone-sensitive lipase (*HSL*) was remarkably downregulated by MC-LR exposure (*p* < 0.05). The 3-hydroxy-3-methylglutaryl-coenzyme A reductase (*HMGCR*) expression was markedly upregulated from 3 d in the treated group (*p* < 0.05). *CYP7A1* and *CYP27A1* transcription levels were significantly downregulated from 3 d onward (*p* < 0.05). The transcription levels of *FXR* and *FGF19* were also notably reduced by MC-LR exposure for 30 d (*p* < 0.05). Meanwhile, the protein expression levels of CD36 and SREBP-1c were markedly upregulated, whereas PPAR-α expression was substantially reduced at both 15 d and 30 d in the MC-LR treatment group (*p* < 0.01) (Figure 3B,C).

### 3.4. Oxidative Stress and Inflammatory Response in Hepatopancreas

**Figure 4:**
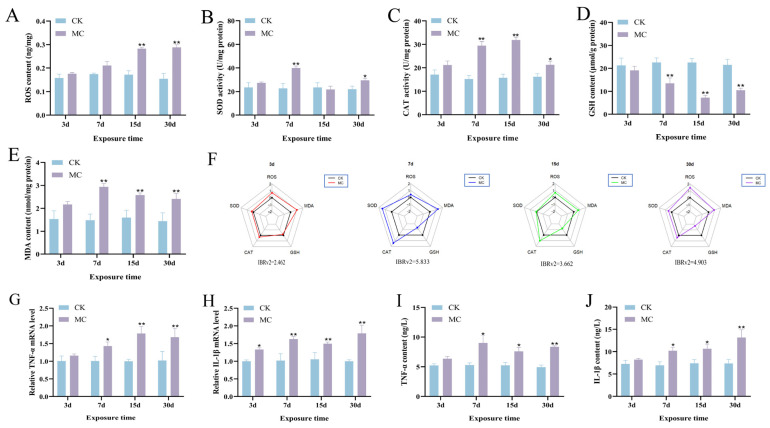
Effect of MC-LR on oxidative stress and inflammatory responses in the hepatopancrea. (**A**) ROS content in the hepatopancreas. (**B**) SOD activity in the hepatopancreas. (**C**) CAT activity in the hepatopancreas. (**D**) GSH content in the hepatopancreas. (**E**) MDA content in the hepatopancreas. (**F**) IBRv2 star plot. In the star plot, each axis represents a specific biomarker, and the distance from the center indicates the relative response level. The overall polygon area reflects the integrated stress response, with larger areas indicating greater physiological disturbance. (**G**) Gene expression of *TNF-α* in the hepatopancreas. (**H**) Gene expression of *IL-1β* in the hepatopancreas. (**I**) TNF-α content in the hepatopancreas. (**J**) IL-1β content in the hepatopancreas. Asterisks denote significant differences between the CK group and the MC group (* *p* < 0.05; ** *p* < 0.01).

The ROS concentration in the hepatopancreas was substantially elevated following MC-LR exposure at 15 d (*p* < 0.01) and 30 d (*p* < 0.01) (Figure 4A). Moreover, SOD activity exhibited a marked elevation at 7 d (*p* < 0.01) and 30 d (*p* < 0.05) after MC-LR exposure (Figure 4B). Similarly, CAT activity was also markedly elevated starting at 7 d (*p* < 0.05) (Figure 4C). In contrast, GSH level was significantly decreased starting from 7 d of MC-LR exposure (*p* < 0.01) (Figure 4D). MDA levels were markedly elevated from 7 d onward (*p* < 0.01) (Figure 4E). The IBRv2 index peaked at 5.833 on 7 d, indicating the highest integrated biological response at this time point (Figure 4F). Changes in TNF-α and IL-1β mRNA expression were largely in line with the alterations seen at the protein level (Figure 4G,H). Specifically, TNF-α content in the hepatopancreas was markedly elevated starting from 7 d under MC-LR exposure (*p* < 0.05) (Figure 4I). Similarly, IL-1β levels showed a significant increase from 7 d onward (*p* < 0.05) (Figure 4J).

### 3.5. Lipidomic Variations of Hepatopancreas

**Figure 5:**
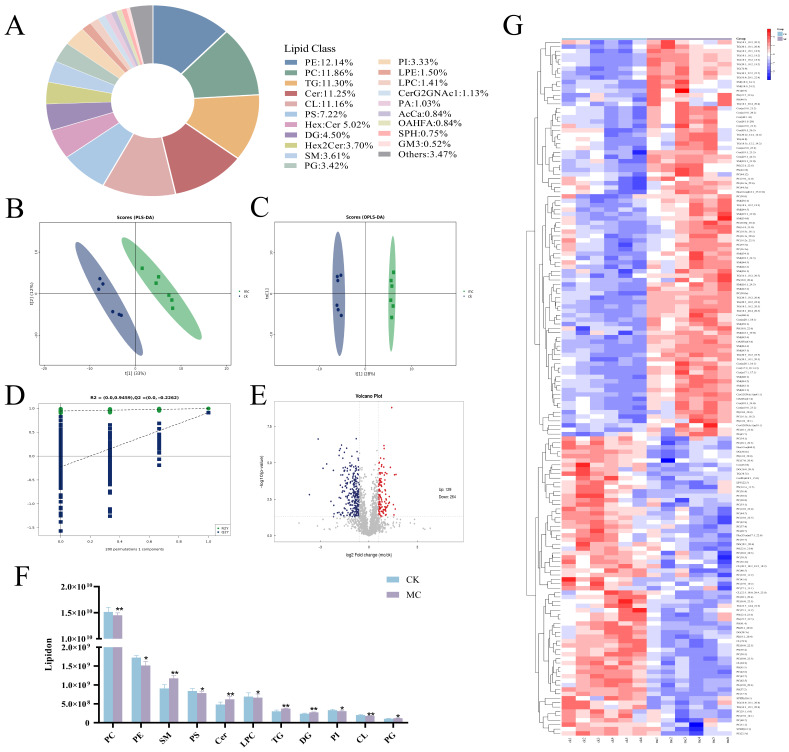
Lipid difference in the hepatopancreas of common carp between the MC group and the CK group at 30 d. (**A**) Pie chart depicting the classification and proportion of lipids. (**B**) PLS-DA model score scatter plot. (**C**) OPLS-DA model score scatter plot. (**D**) Permutation test plot for the OPLS-DA model. (**E**) Volcano plot for CK vs. MC. Red and blue indicate significantly up-regulated and down-regulated lipids, respectively; gray indicates non-significantly different lipids. (**F**) Statistical analysis of the top 11 lipid species with significant differences. (**G**) Heatmap from hierarchical clustering analysis of CK vs. MC. The color blocks represent relative lipid expression, with red indicating high expression and blue indicating low expression. Asterisks denote significant differences between the CK group and the MC group (* *p* < 0.05; ** *p* < 0.01).

Analysis via LC-MS/MS enabled the detection and quantification of 2133 lipid species in the fish hepatopancreas, representing 45 distinct classes (Figure 5A). Among these, phosphatidylethanolamine (PE) was the most abundant lipid, accounting for 12.143% of the total, followed by phosphatidylcholine (PC, 11.861%), triglycerides (TG, 11.299%), and ceramide (Cer, 11.252%). These results indicate that PE, PC, TG, and Cer are the predominant lipid classes in the hepatopancreas, although their relative abundances vary. To investigate lipidomic variations, PLS-DA demonstrated a pronounced group separation (Figure 5B). Orthogonal partial least squares discriminant analysis (OPLS-DA) was subsequently utilized to analyze the lipid composition of all samples, and all data points fell within the 95% confidence interval (Figure 5C). The OPLS-DA model yielded R^2^X, R^2^Y, and Q^2^ values of 0.537, 0.977, and 0.911, respectively, indicating strong explanatory and predictive power. A permutation test was performed to evaluate the model’s robustness and rule out overfitting (Figure 5D). The intercepts for R^2^Y and Q^2^ were 0.946 and −0.226, respectively, confirming the stability and reliability of the model for further analysis. Differential lipids were defined as those meeting the criteria of VIP > 1 and *p* < 0.05. Between the CK and MC group, 393 lipids were differentially expressed, comprising 129 upregulated and 264 downregulated species in the MC group (Figure 5E). Notably, as shown in Figure 5F, glycerolipids such as TG were markedly elevated following MC-LR exposure, while glycerophospholipids including phosphatidylglycerol (PG), PE, and PC were significantly reduced. Additionally, hierarchical clustering heatmaps demonstrated distinct lipidomic profiles between the CK and MC group (Figure 5G).

### 3.6. Correlation Between Altered Lipids and Biochemical Parameters

**Figure 6:**
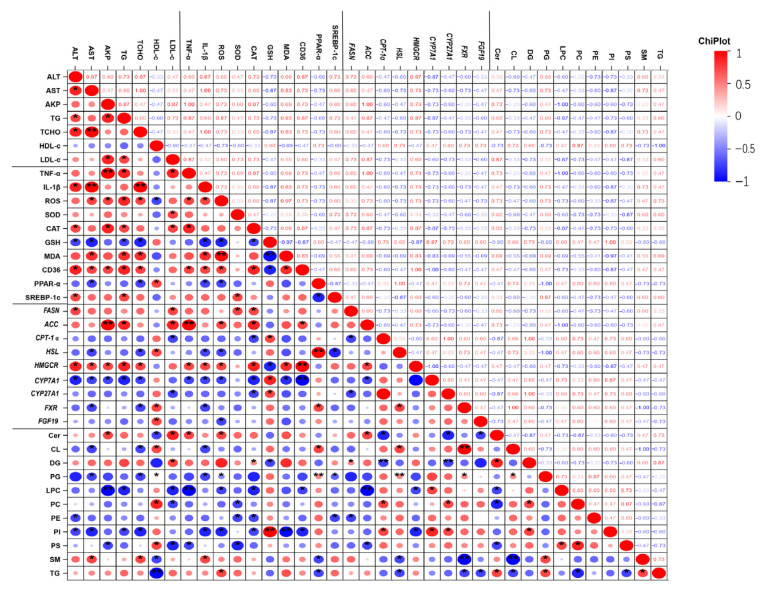
Heatmap of biological parameter correlations after 30 d of MC-LR Exposure. Red for positive correlations, and blue for negative correlations. Asterisks denote significant differences between the CK group and the MC group (* *p* < 0.05; ** *p* < 0.01).

To further clarify how differential lipid metabolites relate to biological metrics, Pearson correlation analysis was employed (Figure 6). The levels of CL, PG, LPC, PC, PE, PI, and PS were negatively correlated with serum ALT, AST, TG, TCHO, and LDL-C, but positively correlated with HDL-C. In contrast, the levels of Cer, DG, SM, and TG were positively associated with serum ALT, AST, TG, TCHO, and LDL-C. Additionally, oxidative stress markers including ROS, SOD, CAT, and MDA were positively correlated with lipogenesis-related genes *FASN* and *ACC*, and negatively correlated with lipid oxidation-related genes *PPAR-α* and *CPT-1α*.

## 4. Discussion

Global temperature fluctuation and the ongoing eutrophication of aquatic ecosystems have led to a marked increase in the occurrence of cyanobacterial blooms [30]. Occurrences of cyanobacterial blooms present serious threats to wild aquatic organisms and exert detrimental impacts on fish in aquaculture systems, resulting in substantial economic losses. MC-LR is a key contributor to these toxic effects [10]. Despite extensive research on MC-LR toxicity, its specific impact on lipid metabolism in farmed fish remains inadequately understood. Therefore, investigating lipid metabolic alterations caused by low-dose MC-LR treatment in common carp using lipidomics, in combination with biochemical and molecular analyses.

The hepatopancreas of fish acts as a pivotal component in chemical metabolism, functioning as the primary site of lipid synthesis and exerting a critical influence on overall energy homeostasis [31]. Serum biochemical parameters serve as essential indicators for assessing the physiological and health status of fish [32]. It has been well established that increased concentrations of transaminase are reliable biomarkers of hepatic injury in different fish species [33]. AKP activity is often measured to reflect alterations in hepatopancreatic function under toxic or stress condition [34]. In this research, 30 d of MC-LR exposure notably elevated serum ALT, AST, and AKP concentrations, indicating potential hepatopancreatic damage. Additionally, serum levels of TG, TCHO, and LDL-C were notably elevated. HDL-C levels were markedly decreased following MC-LR exposure. These alterations conform to earlier studies linking increased TG and TCHO levels to dysregulated lipid metabolism in fish [35]. Moreover, histological examination revealed inflammatory cell infiltration and substantial lipid accumulation in fish hepatopancreas, confirming hepatic inflammation and steatosis induced by MC-LR. Importantly, both the biochemical parameters and histological examination became more pronounced with the extension of exposure time, suggesting that hepatopancreatic injury progresses in a time-dependent manner. Collectively, the results suggest that extended exposure to MC-LR induces hepatopancreatic injury and disrupts lipid metabolic homeostasis in common carp.

FASN catalyzes the formation of long-chain fatty acids, and its overactivation is linked to excessive lipid accumulation [36]. Similarly, ACC is critically involved in the metabolic pathway leading to fatty acid and triglyceride generation [37]. SREBP-1c regulates the transcription of multiple lipogenic enzymes, including FASN and ACC, and is closely linked to triglyceride accumulation in hepatocytes [38]. In this study, MC-LR treatment notably enhanced the expression of key lipogenic genes, including *ACC*, *FASN*, and *SREBP-1c*, and also led to a marked increase in SREBP-1c protein level, indicating enhanced lipid biosynthetic activity. CD36 functions as a transporter protein involved in lipid handling [39]. Our findings revealed a notable enhancement of CD36 following MC-LR exposure, suggesting elevated fatty acid absorption from the circulation. PPAR-α is crucial for lipid metabolism. It promotes fatty acid β-oxidation as well as lipid uptake and lipid droplet turnover. One of its downstream targets is CPT-1α, the key enzyme responsible for the mitochondrial import of fatty acids during β-oxidation [40]. Notably, MC-LR exposure markedly suppressed both PPAR-α gene and protein expression and decreased *CPT-1α* mRNA levels, implying a suppression of mitochondrial β-oxidation and impaired lipid catabolism. HSL serves as an important regulator of lipid degradation in fish and is critical for the hydrolysis of intracellular triacylglycerols [41]. This research demonstrated that MC-LR exposure markedly reduced *HSL* expression, further supporting the disruption of lipid breakdown processes. The results indicate that MC-LR can lead to hepatopancreatic lipid accumulation in common carp. This effect is likely mediated through upregulated lipogenesis, reduced fatty acid β-oxidation, and inhibited lipid catabolism.

The rate-limiting enzyme in cholesterol biosynthesis is encoded by HMGCR. Previous studies have demonstrated that treatment with 30 μg/L of MC-LR in zebrafish markedly upregulates *HMGCR* expression in the hepatopancreas. In contrast, CYP7A1 and CYP27A1 are key enzymes involved in cholesterol catabolism, playing essential roles in regulating cholesterol turnover and maintaining lipid homeostasis [42]. Activation of FXR suppresses CYP7A1 expression, thereby limiting bile acid synthesis to prevent excessive accumulation [43]. FGF19 is another important regulator of cholesterol metabolism that facilitates cholesterol transport, modulates intestinal absorption, and contributes to bile acid homeostasis [44]. Exposure to MC-LR in this study resulted in significant upregulation of *HMGCR* and *FXR* in the hepatopancreas, suggesting enhanced cholesterol biosynthesis and feedback regulation. Conversely, the downregulation of *CYP7A1*, *CYP27A1*, and *FGF19* indicates impaired cholesterol degradation and transport. Furthermore, as the exposure time increased, the cholesterol imbalance became more severe. This result suggests that the accumulation of MC-LR disrupts hepatic lipid metabolism by promoting cholesterol accumulation and inhibiting its clearance.

Oxidative stress is a key factor contributing to hepatic injury in fish, primarily characterized by excessive ROS production and reduced enzymatic antioxidant activity [45]. The decline in antioxidant defense further aggravates ROS accumulation, ultimately leading to hepatopancreatic damage. Among oxidative stress markers, MDA has been widely used as a reliable indicator of lipid peroxidation triggered by ROS [46]. In this study, 30 d of MC-LR exposure caused notable elevations in ROS and MDA, indicating oxidative stress and lipid peroxidation in the hepatopancreas. SOD and CAT serve as primary antioxidant enzymes protecting against oxidative damage and are commonly employed as biomarkers to assess oxidative stress in aquatic organisms. Additionally, GSH plays a pivotal role in detoxification processes by directly scavenging ROS [47]. This result showed that SOD, CAT, and GSH levels were notably decreased following MC-LR exposure, suggesting a compromised antioxidant defense system. Notably, the decline in antioxidant indices was progressive with exposure time, consistent with the aggravated histological deterioration observed in the hepatopancreas. These results collectively indicate that MC-LR exposure induces excessive ROS production, which leads to oxidative imbalance in the hepatopancreas and contributes to hepatic steatosis and functional impairment.

Previous studies have identified oxidative stress as a critical upstream mediator of inflammatory response, and a growing body of evidence has indicated that MC-LR may trigger inflammation in the liver [48,49]. Among inflammatory mediators, TNF-α and IL-1β are key regulators involved in hepatic lipid metabolism [50]. TNF-α exerts its effects by activating multiple signaling pathways through its receptors, thereby influencing genes involved in lipid metabolism [51]. It has been shown to facilitate hepatic fat deposition by enhancing fatty acid synthesis and triglyceride storage. Similarly, IL-1β influences lipid metabolism by initiating inflammatory cascades, primarily by triggering the NF-κB pathway, which regulates hepatic lipid homeostasis [52]. Its sustained activation is closely associated with increased lipogenesis and fat deposition in hepatocytes, potentially aggravating the progression of hepatic steatosis [53]. This study revealed that TNF-α and IL-1β were markedly upregulated at both the gene and protein levels in the hepatopancreas following MC-LR exposure, suggesting that MC-LR induces hepatic inflammation, which may disrupt lipid metabolism and exacerbate liver dysfunction.

Lipidomics is an emerging system biology approach that provides high-throughput and highly sensitive analysis, allowing for comprehensive profiling of lipid species within biological matrices [54]. To clarify how MC-LR affects lipid metabolism in common carp, we performed lipidomic profiling of the hepatopancreas after exposure. OPLS-DA revealed a distinct separation between CK group and MC group, suggesting that MC-LR markedly impacted the hepatopancreatic lipid composition in common carp. Glycerophospholipids are fundamental constituents of cellular membranes. They are crucial in maintaining membrane integrity, facilitating neural development, and regulating lipid metabolism [55,56]. PC is a predominant membrane phospholipid and a key mediator in lipid signaling [57]. Disruption of PC metabolism has been linked to mitochondrial dysfunction, resulting in decreased fatty acid oxidation and diminished energy production [58]. PE is crucial for the production and release of very-low-density lipoproteins (VLDL). Reduced PE levels impair VLDL secretion, disrupt hepatic TG transport [59]. CL is essential for preserving mitochondrial architecture and energetic performance, whereas PG acts as a critical precursor in CL biosynthesis and is abundantly present in the mitochondrial membrane [60]. In this work showed that MC-LR exposure significantly altered the metabolic profiles of PC, PE, CL, and PG, suggesting potential impairments in glycerophospholipid metabolism and organelle membrane function in common carp. Furthermore, Cer function as key lipid mediators participating in metabolic regulation, oxidative responses and diverse pathological processes [61]. Increased Cer levels are associated with lipotoxic effects, inflammatory responses and metabolic dysfunction [62]. These results suggest that MC-LR may disrupt cellular homeostasis and promote inflammatory response in fish hepatopancreas by modulating ceramide metabolism.

MC-LR affects not only the health of individual fish but also the stability of entire aquatic ecosystems. Oxidative stress and inflammatory responses induced by MC-LR may impair immune defenses, increase susceptibility to pathogens, and reduce survival rates [63]. Moreover, liver damage can impair reproductive capacity by disrupting energy supply and lipid and fatty acid metabolism, thereby potentially altering food web structure and compromising ecosystem stability [64]. This study investigated the effects of low-dose MC-LR on lipid metabolism in the carp hepatopancreas. However, the use of a single concentration limits the establishment of a dose–response relationship and the determination of toxicity thresholds. Therefore, further investigations are required.

## 5. Conclusions

Taken together, chronic exposure to low-dose MC-LR induced significant disruption in lipid metabolism in common carp, characterized by oxidative stress, inflammatory response, enhanced lipid synthesis, impaired fatty acid β-oxidation, and cholesterol accumulation. The integration of histological, biochemical, and lipidomics analyses revealed a multifaceted mechanism by which MC-LR compromises hepatopancreatic function. This work provides new insight into the toxicological impact of MC-LR on fish and offers a valuable foundation for future research on lipid metabolism-related pathologies and hepatoprotective strategies in fish.

## Data Availability

Data supporting the reported results are contained within the article.

## Supplementary Materials

The following supporting information can be downloaded at: https://www.mdpi.com/article/10.3390/ani15192803/s1, Table S1: Primer sequences for synthetic genes.
