# Supplementary material for: Alterations in Lipid Metabolism and Hepatopancreatic Lipidomics Induced by Microcystin-LR Exposure in Common Carp (Cyprinus carpio)
Source: Animals (Basel). 2025 Sep 25;15(19):2803. doi: 10.3390/ani15192803 (PMC12523768; doi:10.3390/ani15192803)
Supplement: Supplementary file 1 [file animals-15-02803-s001.zip › animals-3883981-supplementary.pdf]

Supplementary materials caption:

Supplementary Methods 1.

The ALT or AST matrix fluid were transferred into a 96-well plate, the serum samples were also added and maintained at 37 °C for 30 min. Then, the 2, 4-dinitrophenylhydrazine was added into each well and the reactions were conducted for 20 min. NaOH solution was added to stop the reaction, and the activities of ALT or AST were determined at 510 nm, based on the standard curve, and expressed as U/L.

AKP activity was determined as follows: AKP catalyzes the hydrolysis of disodium diphenyl phosphate to release phenol, which reacts with 4-aminoantipyrine and potassium ferricyanide under alkaline conditions to form a red hydrazine derivative. Absorbance was measured at 520 nm, and activity was expressed as U/g protein.

To measure TG and TCHO levels, serum samples were transferred into a 96-well plate, followed by the addition of the corresponding reaction reagents. The mixtures were incubated at 37 °C for 10 minutes to allow enzymatic reactions. Subsequently, chromogenic agents were added and the mixtures were further incubated for color development. Absorbance was measured at 500 nm using a spectrophotometer, and the concentrations of TG and TCHO were calculated based on standard curves and expressed in mmol/L.

The concentrations of HDL-C and LDL-C were determined as follows: Serum samples were transferred into a 96-well plate, and the corresponding reaction reagents were added. The mixtures were incubated at 37 °C for 10 minutes to facilitate enzymatic reactions. Following this, chromogenic agents were introduced, and the mixtures were further incubated to allow color development. Absorbance was measured spectrophotometrically at 600 nm, and the concentrations of HDL-C and LDL-C were determined based on standard curves and expressed in mmol/L.

SOD activity in the hepatopancreas was assessed by monitoring the reduction of 2-(4-iodophenyl)-3-(4-nitrophenyl)-5-(2,4-disulfophenyl)-2H-tetrazolium (WST-1). In brief, 20 μl of hepatopancreas homogenate was added to each well of a 96-well plate, followed by 200 μl of reaction mixture. After incubating at 37 °C for 20 minutes,

absorbance was read at 450 nm using a microplate reader. One unit (U) of SOD activity corresponds to the enzyme amount causing 50% inhibition of the reaction. The results were expressed as U per mg of protein.

CAT activity was measured using the ammonium molybdate method. Ammonium molybdate rapidly halts the decomposition of H_2O_2 by CAT and reacts with the residual H_2O_2 to form a yellow complex. The CAT activity was quantified by measuring absorbance at 405 nm and expressed as U/mg protein, where one unit (U) corresponds to the amount of enzyme that decomposes 1 μmol of H_2O_2 per second.

GSH levels were determined using the 5,5'-dithiobis-(2-nitrobenzoic acid) (DTNB) assay. In brief, hepatopancreas homogenate was treated with sulfosalicylic acid and centrifuged at 3500 g for 10 minutes. The resulting supernatant was then reacted with DTNB for the GSH measurement. Absorbance was recorded at 412 nm, and GSH content was expressed as μmol per gram of protein.

MDA levels were assessed using the thiobarbituric acid (TBA) method. Under acidic conditions, MDA reacts with TBA at 95 °C for 40 minutes, forming a pink MDA-TBA complex. The absorbance of this product was measured at 532 nm, and MDA concentration was expressed as nmol per mg of protein.

Supplementary Methods 2.

The levels of ROS, TNF-α, and IL-1β were measured using commercial ELISA kits. Briefly, plasma or hepatopancreas homogenates were added to 96-well plates pre-coated with specific antibodies and incubated at 37 °C for 30 minutes. After washing the plates five times with wash buffer, horseradish peroxidase (HRP) conjugate was added and incubated under the same conditions. Following another five washes, TMB substrate was added and the reaction was allowed to proceed in the dark for 10 minutes. The reaction was then stopped with a termination solution, and absorbance was measured at 450 nm using a microplate reader (Multiskan Sky, Thermo Fisher Scientific, Waltham, MA, USA). Concentrations were calculated based on standard curves and expressed accordingly.

Table S1. Primer sequences for synthetic genes

Primers	Forward (5′-3′)	Reverse (5′-3′)	Accession number
CD36	GGTGCAGAAAGGACCGTACA	CCTACGGACATGCTAGGCTC	XM_059511931.1
PPAR-α	ATGAGGCTAAGAAGCGGACG	CATCCAAAGTCCCCGAACCA	XM_042741297.1
SREBP-1c	GCCGCAGTTCATTAAGGCAG	GGATGTAGCCAGCGATGTGA	XM_019073316.1
ACC	ACGCCCCTGCATCATCATAG	ATAACATCACAGCACCGCCA	XM_042723589.1
FASN	TCCCTTCCAATCCCAACGTG	GGGGTACAAACCAGGGATGG	XM_019070657.1
CPT-1α	GTTCATCTCGCTGGGAGGAG	TCTCTGTGCAGGAGTGCTTG	XM_019122018.1
HSL	ACAAGCAGGTTAGGGCGAAA	CACCGCATATCGAGCAGACT	XM_042776498.1
HMGCR	GGTTGCTGGCCCTCTGTTAT	CATCTGCAAGAACACGGCTG	XM_019073034.1
CYP7A1	AGACCAGAGTATTGCACGCC	CCAGCGATCAGAGCAGGAAA	XM_019074263.1
CYP27A1	AGATGTGGTCAATGCGGT	AGCCAATGCGTGTCTCAA	XM_019102935.1
FXR	GGTGCTGAGGAAGGTCTG	GTGGTGGTGGTTGAGGGT	XM_019069273.1
FGF19	TGTGATCCAGAGTCCCAG	GTGTCCCGTGTGCAGTTT	XM_019066901.1
IL-1β	GCTGGAGCAATGCAATACAAA	AGGTAGAGGTTGCTGTTGGAA	AB010701.1
TNF-α	ACAACAATCAGGAAGGTGGAA	TGGAAAGACACCTGGCTGTA	XM_019088899.1
β-actin	GCTATGTGGCTCTTGACTTCG	CCGTCAGGCAGCTGATAGCT	JQ619775.1
